# Higher lipid turnover and oxidation in cultured human myotubes from athletic versus sedentary young male subjects

**DOI:** 10.1038/s41598-018-35715-7

**Published:** 2018-12-03

**Authors:** Jenny Lund, Siw A. Helle, Yuchuan Li, Nils G. Løvsletten, Hans K. Stadheim, Jørgen Jensen, Eili T. Kase, G. Hege Thoresen, Arild C. Rustan

**Affiliations:** 1 Department of Pharmaceutical Biosciences, School of Pharmacy, University of Oslo, Oslo, Norway; 2 Department of Nutrition, Institute of Basic Medical Sciences, University of Oslo, Oslo, Norway; 3 Department of Physical Performance, Norwegian School of Sport Sciences, Oslo, Norway; 4 Department of Pharmacology, Institute of Clinical Medicine, University of Oslo, Oslo, Norway

## Abstract

In this study we compared fatty acid (FA) metabolism in myotubes established from athletic and sedentary young subjects. Six healthy sedentary (maximal oxygen uptake (VO_2max_) ≤ 46 ml/kg/min) and six healthy athletic (VO_2max_ > 60 ml/kg/min) young men were included. Myoblasts were cultured and differentiated to myotubes from satellite cells isolated from biopsy of *musculus vastus lateralis*. FA metabolism was studied in myotubes using [^14^C]oleic acid. Lipid distribution was assessed by thin layer chromatography, and FA accumulation, lipolysis and re-esterification were measured by scintillation proximity assay. Gene and protein expressions were studied. Myotubes from athletic subjects showed lower FA accumulation, lower incorporation of FA into total lipids, triacylglycerol (TAG), diacylglycerol and cholesteryl ester, higher TAG-related lipolysis and re-esterification, and higher complete oxidation and incomplete β-oxidation of FA compared to myotubes from sedentary subjects. mRNA expression of the mitochondrial electron transport chain complex III gene *UQCRB* was higher in cells from athletic compared to sedentary. Myotubes established from athletic subjects have higher lipid turnover and oxidation compared to myotubes from sedentary subjects. Our findings suggest that cultured myotubes retain some of the phenotypic traits of their donors.

## Introduction

Skeletal muscle cells store glucose as glycogen and fatty acids (FAs) in triacylglycerol (TAG)-containing lipid droplets (LDs)^1–6^. It has previously been described that exercise may change the LDs morphology, composition, localization, and mobilization^7^. Perilipins (PLINs) are LD-associated proteins that amongst others determine size and stability of the LDs, level of interaction between LDs and mitochondria as well as regulation of lipolysis, FA oxidation and cellular levels of TAG^8,9^. It has been hypothesized that there is a better interaction between LDs and mitochondria in endurance-trained subjects than sedentary untrained^10^. This is in coherence with the theory of faster intramyocellular triacylglycerol (IMTG) turnover in endurance-trained subjects^11^. The oxidative capacity of myotubes, lipolysis, availability of FAs as well as re-esterification of lipid intermediates, *e.g*. diacylglycerol (DAG), are all factors of significance for the dynamic IMTG turnover rate^12,13^.

Metabolic capacity in skeletal muscles during exercise varies greatly between sedentary and athletic subjects. Athletic subjects have higher capacity for energy production, and particularly increased lipid oxidation at the expense of carbohydrate oxidation^14,15^. An earlier report showed that with increasing delivery of free fatty acids (FFAs) to muscle, plasma FFA uptake and oxidation increased in trained subjects during prolonged thigh exercise, whereas untrained reached a plateau^16^. In general, it has been suggested that skeletal muscle of endurance-trained subjects has a higher capacity for oxidative metabolism as opposed to sedentary subjects^17^.

We have previously described, using cultured skeletal muscle cells from the same athletic (maximal oxygen uptake, VO_2max_ > 60 ml/kg/min) and sedentary (VO_2max_ ≤ 46 ml/kg/min) subjects as in the current work, that myotubes from athletic subjects showed higher deoxyglucose accumulation and fractional glucose oxidation. Further, myotubes from athletic donors were also more sensitive to the suppressive action of acutely added oleic acid to the cells^18^.

The purpose of this study was to examine FA uptake, esterification, turnover, and oxidation in myotubes established from athletic subjects (maximal oxygen uptake, VO_2max_ > 60 ml/kg/min) compared to cells from sedentary subjects (VO_2max_ ≤ 46 ml/kg/min).

## Results

### Donor characteristics and performance parameters

Selected donor characteristics are presented in Table 1, and have been presented in full previously^18^. As expected the athletic subjects performed at a higher workload_max_ and they also had higher maximal fat oxidation *in vivo*, as determined during an incremental test, compared to sedentary subjects (Table 1).

**Table 1:** Clinical parameters.

	Sedentary	Athletic
n	6 (^a^4)	6
Age, y	26.8 ± 2.8	24.3 ± 0.7
Body weight, kg	93.5 ± 8.8	81.4 ± 2.9
BMI, kg/m^2^	28.0 ± 2.5	23.9 ± 0.9
WHR	0.91 ± 0.02^a^	0.89 ± 0.01
VO_2max_, ml/kg/min	41.1 ± 1.6	64.9 ± 1.7*
Workload_max_, watt	255.0 ± 27.9	287.5 ± 14.1*
Fat oxidation_max_, mg/min/kg body weight	3.7 ± 0.5	5.1 ± 1.1*

Fat oxidation rates were calculated from measured oxygen uptake and carbon dioxide output using Frayn’s equations^54^. Maximal fat oxidation independent of exercise intensity was chosen. Values are given as means ± SEM, and n represents the number of subjects. *Statistically significant versus sedentary (*p* < 0.05, Mann-Whitney test). ^a^Missing data from two participants. BMI, body mass index; VO_2max_, maximal oxygen uptake; WHR, waist-to-hip ratio. Complete set of donor characteristics have been reported in full previously^18^.

### Lower FA accumulation and incorporation into complex lipids in myotubes from athletic subjects

**Figure 1:**
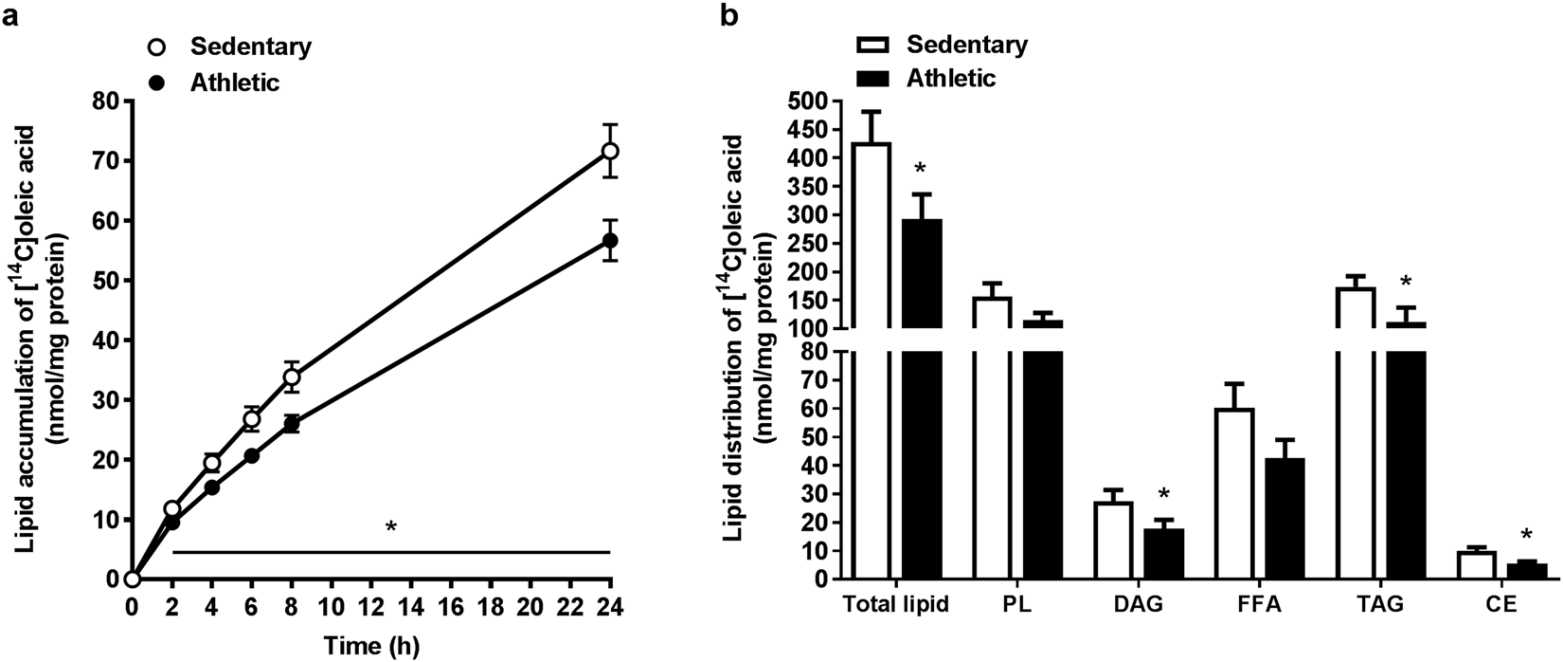
Lower oleic acid accumulation and incorporation into lipids in myotubes from athletic subjects. Satellite cells isolated from biopsies from *musculus vastus lateralis* from athletic and sedentary subjects were cultured and differentiated into myotubes. **(a)** Fatty acid accumulation of [^14^C]oleic acid (100 µM) during the last 24 h of the differentiation period was measured with SPA. (**b)** Myotubes were treated with [^14^C]oleic acid (100 µM) for 24 h, and lipids were thereafter extracted from the cells, separated by TLC and quantified by liquid scintillation. CE, cholesteryl ester; DAG, diacylglycerol; FFA, free fatty acid; PL, phospholipid; TAG, triacylglycerol. Data are presented as means ± SEM (n = 6 in each group) in nmol/mg cell protein. *Statistically significant versus sedentary (*p* < 0.05, a: Linear mixed-model analysis, SPSS, b: Mann-Whitney test).

Myotubes were treated with [^14^C]oleic acid (100 µM) the last 24 h of the differentiation period. FA accumulation over the 24 h was lower in myotubes from athletic subjects compared to myotubes from sedentary (Fig. 1a). Furthermore, lipid distribution after the 24 h showed that myotubes from athletic subjects had reduced incorporation of [^14^C]oleic acid into DAG, TAG and cholesteryl ester (CE), as well as total cellular lipids compared to myotubes from sedentary subjects (Fig. 1b).

LDs (illustrated in Supplementary Fig. S1a) were isolated from myotubes from a limited number of donors, after treating the myotubes with 100 µM oleic acid during the last 24 h of the differentiation period. There were no differences in TAG mass in isolated LDs from myotubes between the two groups (Supplementary Fig. S1b). Number of LDs related to TAG mass of the isolated LDs (Supplementary Fig. S1c) was not different between the two groups. In total, 45 subspecies of TAGs were detected (Supplementary Fig. S1d) from isolated LDs. Two subspecies were particularly abundant, TAG(52:2) and TAG(54:3). However, no differences in TAG subspecies were found between cells from the two donor groups.

In cultured human myotubes, PLIN2 and PLIN3 are the most abundantly expressed PLINs^19^. Neither mRNA or protein expression of the LD-coating proteins PLIN2 and PLIN3 showed differences between the two groups (Supplementary Fig. S1e–g).

### Higher TAG-related lipolysis and re-esterification in myotubes from athletic subjects

**Figure 2:**
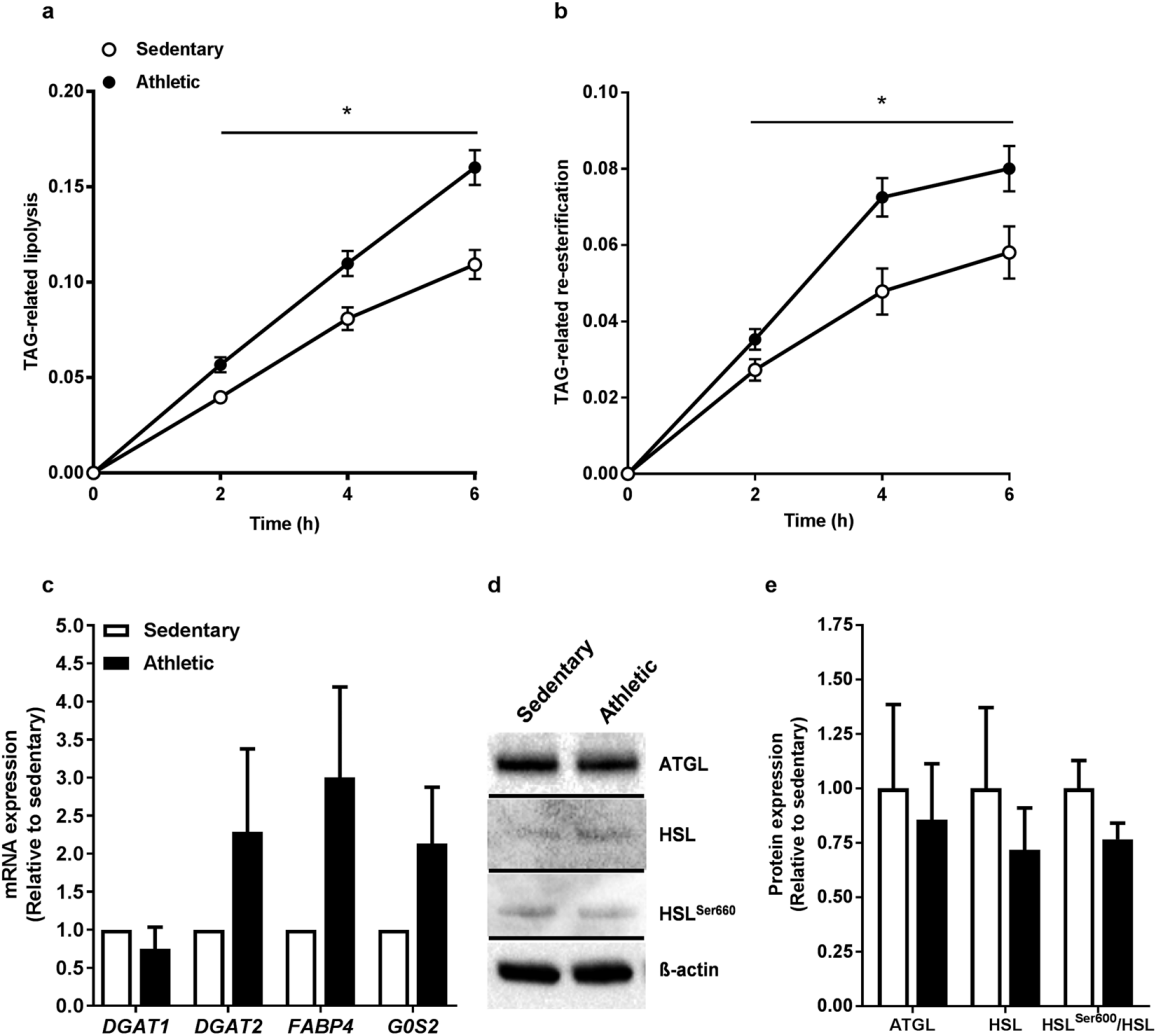
Higher TAG-related lipolysis and re-esterification in myotubes from athletic subjects. (**a)** Lipolysis was measured over 6 h after 24 h incubation with [^14^C]oleic acid. Lipolysis was related to the labelled triacylglycerol (TAG) pool in the cells as measured by TLC. **(b)** Re-esterification was measured over 6 h after 24 h incubation with [^14^C]oleic acid. Re-esterification of oleic acid was calculated from lipolysis measurements as [triacsin C present (total lipolysis) – triacsin C absent (basal lipolysis)]. Fatty acid re-esterification was related to the labelled TAG pool in the cells as measured by TLC. Data are presented as means ± SEM (n = 6 in each group). *Statistically significant versus sedentary (*p* < 0.05, linear mixed-model analysis, SPSS. **(c)** RNA was isolated and mRNA reversely transcribed before expressions of diacylglycerol acyltransferase (*DGAT*) *1*, *DGAT2*, fatty acid binding protein (*FABP*) *4*, and G0/G1 switch gene 2 (*G0S2*) were assessed by qPCR. Values are presented as means ± SEM (n = 6 in each group), and corrected for the average of the housekeeping gene acidic ribosomal phosphoprotein P0 (*RPLP0*). The results were normalized to the results of the mRNA expression for myotubes from sedentary subjects. **(d,e)** Protein expressions of adipose triglyceride lipase (ATGL), hormone-sensitive lipase (HSL) and phosphorylated HSL at serine 660 (HSL^Ser660^) were analysed by immunoblotting of protein isolated from cell lysates. d, representative immunoblots. e, quantified expressions of the proteins. All values were corrected for the housekeeping control β-actin, and are presented as means ± SEM (n = 6 in each group). All samples were derived at the same time, from the same experiment, and processed in parallel. Full-length blots are presented in Supplementary Fig. S2.

After 24 h accumulation of [^14^C]oleic acid, lipolysis and re-esterification were measured over 6 h. These processes are presented in Fig. 2a,b related to the labelled TAG pool in the myotubes. We observed that cells from athletic subjects had higher lipolysis than cells from sedentary subjects (Fig. 2a). Moreover, a higher FA re-esterification in myotubes from athletic subjects was also seen (Fig. 2b). However, mRNA levels of the TAG synthesizing enzymes diacylglycerol (*DGAT) 1* and *DGAT2*, fatty acid binding protein (*FABP*) *4* and G0/G1 switch gene 2 (*G0S2*) (Fig. 2c), as well as protein expression of the lipolytic pathway proteins adipose triglyceride lipase (ATGL) and hormone-sensitive lipase (HSL) revealed no differences between cells from the two groups (Fig. 2d,e). Phosphorylation of HSL was also not different between the groups (Fig. 2d,e).

**Figure 3:**
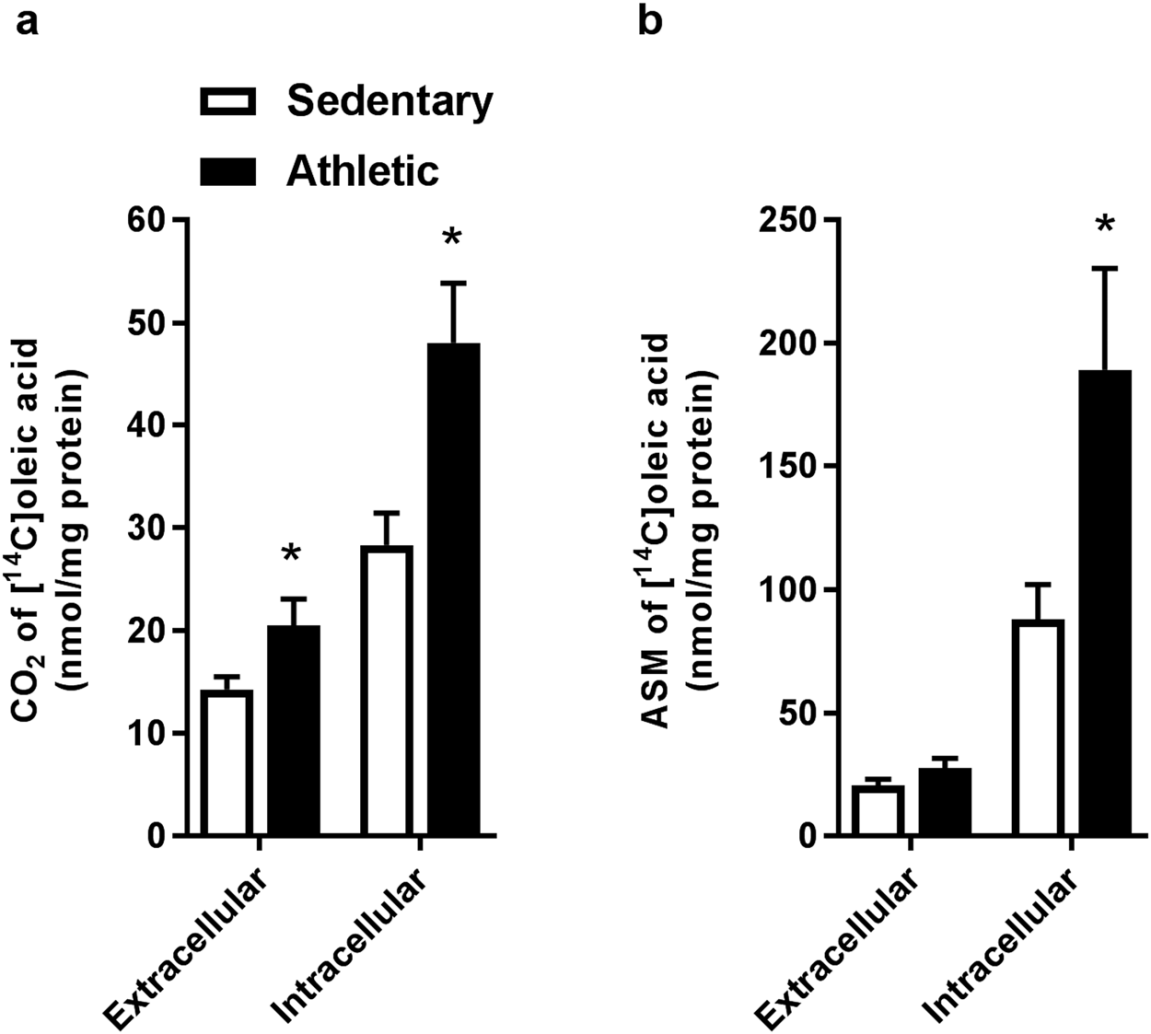
Higher oleic acid metabolism in myotubes from athletic subjects. Satellite cells isolated from biopsies from *musculus vastus lateralis* from athletic and sedentary subjects were cultured and differentiated into myotubes. CO_2_ (complete fatty acid oxidation) and acid-soluble metabolites (ASMs, representing incomplete fatty acid β-oxidation) were measured after extracellular treatment (100 µM [^14^C]oleic acid for 4 h) and intracellular treatment (prelabelling with a combination of 100 µM and 400 µM [^14^C]oleic acid for 24 h). **(a)** CO_2_ from extracellular (acute) and intracellular (prelabelled) [^14^C]oleic acid. **(b)** ASM from extracellular and intracellular [^14^C]oleic acid. Values are presented as means ± SEM (n = 6 in each group) in nmol/mg cell protein. *Statistically significant versus sedentary (*p* < 0.05, a: Mann-Whitney test, b: Linear mixed-model analysis, SPSS).

### Higher oleic acid oxidation from extracellular and intracellular oleic acid in myotubes from athletic subjects

To further examine FA metabolism we measured complete oxidation and incomplete β-oxidation in the myotubes after acute (4 h) and chronic (24 h) incubation with [^14^C]oleic acid (Fig. 3). Cells from athletic subjects showed higher complete oxidation (CO_2_ production, Fig. 3a) from both extracellular (acute) and intracellular (prelabelled) [^14^C]oleic acid, as well as higher incomplete FA β-oxidation (acid soluble metabolites, ASMs, Fig. 3b) from intracellular oleic acid compared to cells from sedentary subjects.

### Minor differences in mRNA expression in myotubes from the two groups

**Figure 4:**
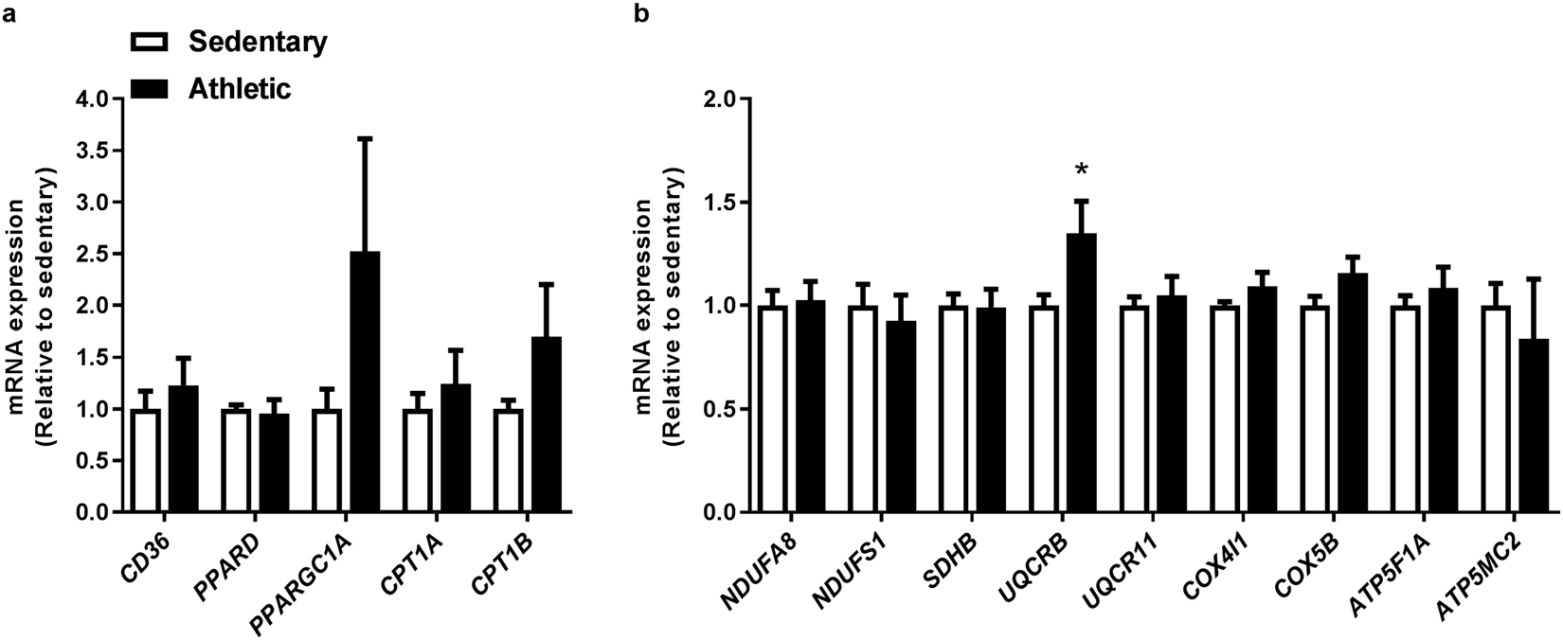
Minor differences in mRNA expression in myotubes from the two groups. Satellite cells isolated from biopsies from *musculus vastus lateralis* from athletic and sedentary subjects were cultured and differentiated into myotubes. RNA was isolated and mRNA reversely transcribed before expressions of selected genes were assessed by qPCR. Values are presented as means ± SEM (n = 6 in each group), and corrected for the average of the housekeeping gene acidic ribosomal phosphoprotein P0 (*RPLP0*). The results were normalized to the results of the mRNA expression for myotubes from sedentary subjects. **(a)** mRNA expression of FA utilization. **(b)** mRNA expression of genes encoding for enzymes of the electron transport chain which drives the oxidative phosphorylation. *Statistically significant versus sedentary (*p* < 0.05, Mann-Whitney test). *ATP5F1A*, ATP-synthase F1 subunit α; *ATP5MC2*, ATP-synthase membrane subunit c locus 2; *CD36*, fatty acid translocase; *COX4I1*, cytochrome c oxidase subunit 4I1; *COX5B*, cytochrome c oxidase subunit 5B; *CPT1A*, carnitine palmitoyltransferase 1A; *CPT1B*, carnitine palmitoyltransferase 1B; *NDUFA8*, NADH-ubiquinone oxidoreductase core subunit A8; *NDUFS1*, NADH-ubiquinone oxidoreductase core subunit S1; *PLIN2*, perilipin 2; *PLIN3*, perilipin 3; *PPARD*, peroxisome proliferator-activated receptor δ; *SDHB*, succinate dehydrogenase complex iron sulphur subunit B; *UQCR11*, ubiquinol-cytochrome c reductase complex III subunit XI; *UQCRB*, ubiquinol-cytochrome c reductase binding protein.

To probe mechanisms behind the difference in FA oxidation between cells from the two donor groups we measured mRNA expression of selected genes involved in FA utilization, including genes encoding for enzymes of the electron transport chain which drives the oxidative phosphorylation (Fig. 4). mRNA expression of fatty acid translocase (*CD36*), an important transporter of FAs across the plasma membrane^20,21^, was not different between cells from the two groups (Fig. 4a). The expression of *PPARD* was also not different between the two groups. Furthermore, expression of peroxisome proliferator-activated receptor γ coactivator 1α (*PPARGC1A*), which codes for the master regulator of mitochondrial biogenesis PGC-1α^7,22,23^, as well as expressions of the carnitine palmitoyltransferases (*CPT*) *1A* and *1B* (Fig. 4a), genes coding for proteins involved in FA transport into mitochondria^24^, was not different between the two groups either.

For genes involved in oxidative phosphorylation, mRNA expressions of NADH-ubiquinone oxidoreductase (*NDUF*) subunits *A8* and *S1* (complex I), succinate dehydrogenase complex iron sulphur subunit B (*SDHB*, complex II), cytochrome c oxidase (*COX*) subunits *4I1* and *5B* (complex IV), and ATP-synthase F1 subunit α (*ATP5F1A*) and ATP-synthase membrane subunit c locus 2 (*ATP5MC2*), both complex V, were not different between the cells from the two groups (Fig. 4b). With regards to complex III, the expression of ubiquinol-cytochrome c reductase binding protein (*UQCRB*) was higher in cells from the athletic compared to cells from the sedentary, but there were no differences in mRNA expression of ubiquinol-cytochrome c reductase complex III subunit XI (*UQCR11*) between cells from the two groups (Fig. 4b).

## Discussion

In this study we showed that myotubes established from young athletic subjects accumulated less oleic acid compared to myotubes from sedentary subjects. In line with this, myotubes from athletic subjects had lower FA incorporation into DAG, TAG and CE, as well as lower overall incorporation into complex lipids than myotubes from sedentary subjects. Moreover, FA lipolysis and re-esterification were higher in myotubes from athletic subjects when related to the labelled cellular TAG pool. Finally, our results also showed that myotubes from athletic had a higher oleic acid oxidation compared to cells from sedentary subjects.

Lipid metabolites rather than the total lipid content have been proposed to be a major offender in development of insulin resistance in skeletal muscle^25,26^. Elevated levels of lipid metabolites such as DAGs and ceramides have been observed in skeletal muscle from obese and subjects with type 2 diabetes mellitus^25,26^, and it is proposed that ceramide, DAG and long-chain FA acyl-CoA may interfere with insulin signalling^11,27,28^. On the other hand, it has been shown that endurance-trained athletes and insulin sensitive subjects with high levels of IMTG also accumulate DAG, thus it is possible that various composition of lipid intermediates leads to different regulations^8^. In the present study we observed lower FA accumulation in myotubes from athletic subjects compared to sedentary. Moreover, myotubes from athletic subjects did in fact have lower overall FA incorporation into complex lipids, with lower incorporation into total lipids as well as reduced DAG and CE in addition to lower TAG incorporation. It thus seems that myotubes from athletic subjects were better in shuffling oleic acid towards FA oxidation, *i.e*. myotubes from athletic expressed a different lipid dynamics. In line with this, a decrease in DAGs and ceramide has been found in skeletal muscle biopsies after exercise^29^, fitting with the results from our study where we observed lower incorporation of oleic acid into DAG.

As LDs are dynamic storage organelles for TAG and other neutral lipids in skeletal muscle^3^, understanding the regulation and composition of LDs would likely be an integral part to understand overall skeletal muscle lipid metabolism. TAG composition of isolated LDs was examined in a small subset of donors, however no differences were observed between the two groups. Different PLINs may have distinct roles in TAG management^3^. Protein expression of PLIN2 has been shown to be decreased with endurance training in lean men^27^, whereas protein expression of PLIN5 has been shown to be higher in endurance-trained compared to sedentary subjects^6^. Protein expressions of PLIN3 and PLIN4 have on the other hand been shown to be unaffected by endurance training^27^. We did however not observe any differences in expression of the most dominant PLINs (PLIN2 and PLIN3) in cultured skeletal muscle cells, neither on mRNA nor on protein level.

Lipolysis is the biochemical pathway responsible for catabolism of TAG stored in LDs^30^. It has been shown that PLIN2 limits ATGL binding to LDs and slows TAG turnover^26^. Lipolysis may mediate formation of lipid intermediates^31^. Lipolysis and re-esterification related to the level of labelled TAG were markedly higher in myotubes from athletic compared to sedentary subjects, suggesting a higher turnover of LDs. It has been observed that TAG/FA cycling, a futile cycle where esterification of TAG is followed by hydrolysis, plays an important role in controlling lipid metabolism *in vivo* in humans during and after exercise^32,33^. In skeletal muscle it has previously been accepted that HSL is the principal enzyme responsible for lipolysis of IMTG during exercise^28–30,34^; however, more recently ATGL has emerged as the major regulator in lipolysis of IMTG during contractile activity^35,36^. Upon energy demand, *e.g*. during exercise, the enzymatic degradation of the esterified neutral lipids in the LD core into single lipid species depends on active recruitment of these lipases to the LD surface. ATGL is considered the first step in catabolism of TAG^37^. This generates DAG; which is further degraded by HSL^38^. Thus, to further look for possible mechanistic explanations for higher lipid turnover, we measured expression of enzymes important for regulating lipolysis in human myotubes. We did not observe any differences in protein expressions of ATGL or HSL. Regulation of ATGL depends on co-regulators like comparative gene identification-58 (CGI-58)^34,39^ and G0S2^40,41^, which are co-activator and inhibitor of ATGL, respectively. We did not observe any significant difference in mRNA expression of *G0S2* in our study. Studies in human myotubes have clearly shown the limiting role of CGI-58 in the control of lipolysis and ATGL activity^39^. Furthermore, ATGL and CGI-58 have been shown to be strongly associated during contraction-induced lipolysis and to work together with PLINs to regulate lipolysis^42^. Activity of the HSL protein is mostly regulated by phosphorylation, such as phosphorylation on Ser660^39^. Thus, we also examined the protein expression of Ser660-phosphorylated HSL; no difference was observed between the two groups. However, the HSL protein needs to translocate from the cytoplasm, thus the full functional picture of this enzyme may also not be revealed from studying protein level^35,36^. FABP4 has been shown to interact with HSL to enhance lipolysis^43,44^. *FABP4* mRNA expression was not different between cells from the two groups in our study (*p* = 0.08). Further research may be needed to explain the functional data observed in this study.

Our results showed that athletic subjects had a higher maximal fat oxidation *in vivo* compared to sedentary. In the myotubes from athletic individuals we also observed a higher extracellular (acute) complete oleic acid oxidation. Furthermore, by studying FA oxidation after prolonged treatment with higher concentrations of [^14^C]oleic acid (*i.e*. intracellular FA oxidation), we observed that not only complete FA oxidation was higher, but also incomplete FA β-oxidation (ASMs) in myotubes from athletic but not in myotubes from sedentary subjects. Unfortunately, the biopsy material in this study was limited, not allowing us to study lipid content and metabolism *ex vivo*. However, other studies *in vivo*^31^ and *ex vivo*^45^ have shown similar effects of endurance-training on FA oxidation after infusion or incubation with FAs, respectively, which indicates that this property may be conserved *in vitro*. Taken together, our findings indicate that skeletal muscle cells from athletic subjects to a greater extent than sedentary are able to regulate FA metabolism as a result of an increased availability of FAs.

We have previously reported that there were no differences in mRNA expression of cytochrome c-1 or protein expressions of mitochondrial oxidative phosphorylation proteins in myotubes from the same sedentary and athletic subjects^18^. Mostly there were no significant differences in gene expression between cells from the two groups; however, mRNA expression of *UQCRB* was higher in cells from athletic compared to cells from sedentary subjects. It has been observed in skeletal muscle biopsies that mRNA expression of *UQCRB* is a very strong predictor for VO_2max_; the higher expression of *UQCRB* the higher VO_2max_^46^. An earlier report showed that acute exercise increased mRNA expression of *PPARGC1A* in skeletal muscle^47^, which has also been shown after chronic exercise *ex vivo*^48^, but we were not able to confirm this in our study with myotubes.

In our study, both genetic differences and acquired changes, from *e.g*. lifestyle and/or training, may possibly contribute to the observed differences between the donor groups. However, studies examining effects of exercise *in vivo* on energy metabolism *in vitro* has shown that training *in vivo* is able to induce changes in human myotubes that are discernible *in vitro*, as indicated by both increased glucose and lipid metabolism in the cultured myotubes^49,50^. In addition, by using electrical pulse stimulation on cultured myotubes from healthy donors we have previously shown increased oxidative capacity by increasing glucose metabolism and fatty acid oxidation^51,52^. Thus, exercise-induced changes may be responsible for the essence of our findings. Combining the results from the current work with the previous report on the same cells^18^, we see that myotubes from athletic donors show higher metabolic capacity, especially for FAs but also with regards to glucose. Furthermore, we have observed that the cells from athletic subjects are more metabolically flexible^18^.

In conclusion, this study showed that differentiated skeletal muscle cells from athletic subjects had a higher FA turnover and oxidation compared to myotubes from sedentary subjects, indicating a higher preference for FA metabolism with better training status. Thus, cultured myotubes from athletic subjects appear to have different metabolic properties than myotubes from sedentary, indicating that they retain some of the exercise-induced characteristics of their donors despite the fact that the satellite cells do not undergo muscle contractions themselves. This is in coherence with a previous report using cells from the same donors, which showed that the myotubes retained some of their phenotypic traits *in vitro* with respect to higher glucose metabolism^18^.

## Materials and Methods

### Materials

96-well Corning^®^ CellBIND^®^ tissue culture plates were from Corning (Schiphol-Rijk, the Netherlands). Nunc™ Cell Culture Treated Flasks with Filter Caps, Nunc™ 96-Well Polystyrene Conical Bottom MicroWell™ Plates, Nunc™ 96-MicroWell™ plates, Bodipy 493/503, Hoechst 33258 and 33342, antibodies against ADFP (#PA5-25042)^53^, TIP47 (#PA5-20272)^53^ and HSL phosphorylated at serine 660 (#PA5-64494)^53^, TaqMan reverse transcription kit reagents, MicroAmp^®^ Optical 96-well Reaction Plate, MicroAmp^®^ Optical Adhesive Film, High-Capacity cDNA Reverse Transcription Kit, Primers for TaqMan PCR, Power SYBR^®^ Green PCR Master Mix, DMEM-Glutamax™ low glucose with sodium pyruvate, DMEM without phenol red, FBS, Trypsin-EDTA, penicillin-streptomycin (10,000 IE/ml), amphotericin B, and DPBS (without Mg^2+^ and Ca^2+^) were from Thermo Fisher Scientific (Waltham, MA, US). Trypan blue 0.4% solution, DMSO, L-glutamine, BSA (essentially FA-free), L-carnitine, D-glucose, oleic acid (18:1, n-9), HEPES, protease inhibitor, phosphatase II inhibitor, cOmplete™ Protease Inhibitor Cocktail, β-mercaptoethanol, and Triacsin C were from Sigma-Aldrich (St. Louis, MO, US). Ultroser G was from Pall (Cergy-Saint-Christophe, France) and insulin (Actrapid^®^ Penfill^®^ 100 IE/ml) from Novo Nordisk (Bagsvaerd, Denmark). SkBM-kit (SkGM) and BioWhittaker^®^ PBS were from Lonza (Wakersville, MD, US). OptiPhase Supermix, 96-well Isoplate^®^, Unifilter^®^-96 GF/B, and TopSeal^®^-A transparent film were from PerkinElmer (Shelton, CT, US). [1-^14^C]oleic acid (56.3 mCi/mmol) was from PerkinElmer NEN^®^ (Boston, MA, US). Clarity™ Western ECL Substrate, Tris/glycine buffer, Tris/glycine/SDS buffer, SDS, Tween 20, bromophenol blue, goat anti-rabbit IgG (H+L)-HRP Conjugate (#170-6515), Mini-Protean^®^ TGX™ gels (4-20%), and Bio-Rad Protein Assay Dye Reagent Concentrate were from Bio-Rad (Copenhagen, Denmark). Glycerol, Tris-HCl and thin layer chromatography plates were from Merck (Darmstadt, Germany). Glass Bottom Microwell Dishes (35 mm petri dish, 14 mm Microwell, No. 1.5 coverglass) were from MatTek (Ashland, MA, US). TG PAP 150-kit was from BioMerieux (Marcy l’Etoile, France). VWR^®^ Grade 703 Blotting Paper was from VWR (Poole, UK). Amersham™ Protran™ Premium 0.45 µm NC Nitrocellulose Blotting Membranes were from Amersham™ (GE Healthcare, Esbjerg, Denmark). Antibodies against HSL (#4107)^53^, ATGL (#2138)^53^ and β-actin (#4970)^18,53^ were from Cell Signaling Technology Inc. (Beverly, MA, US). RNeasy Mini Kit was from QIAGEN (Venlo, the Netherlands).

### Ethics statement

The biopsies were obtained after informed written consent and approval by the Regional Committee for Medical and Health Research Ethics South East, Oslo, Norway (reference number: 2011/2207). The study adhered to the Declaration of Helsinki.

### Donor characteristics

To participate in the study the participants had to be male, 21–37 years old, non-smokers, not use any medicines, and either have a high training status defined as VO_2max_ > 60 ml/kg/min (athletic subjects) or low training status defined as VO_2max_ ≤ 46 ml/kg/min (sedentary subjects). Six men were included in each group. The athletic subjects in our study were not solely endurance-trained athletes, they were students at the Norwegian School of Sport Sciences and performed a range of different sport activities; this has been described previously^18^. Measurement of VO_2max_ was performed on Monark Ergomedic 839E cycle (GIH, Stockholm, Sweden), and workload_max_ was established after an incremental test. *In vivo* fat oxidation was calculated based on VO_2_ and VCO_2_ rates obtained during the incremental test using Frayn’s equations^54^. Maximal fat oxidation independent of exercise intensity was used. Complete set of donor characteristics have been reported previously^18^.

### Culturing of human myotubes

Multinucleated human myotubes were established by activation and proliferation of satellite cells isolated from a small biopsy (100–200 mg) of *musculus vastus lateralis* from six sedentary men and from six athletic men. Isolation of satellite cells was based on the method of Henry *et al*.^55^, modified according to Gaster *et al*.^56,57^, and was performed at the same location by the same trained researchers.

For proliferation of myoblasts DMEM-Glutamax^TM^ (5.5 mM glucose) medium supplemented with FBS (2%) and Ultroser G (2%) was used. At approximately 80% confluence the culture medium was changed to DMEM-Glutamax^TM^ (5.5 mM glucose) supplemented with FBS (2%) and insulin (25 pM) to initiate differentiation into multinucleated myotubes. The cells were allowed to differentiate for seven days; the cells from both donor groups differentiated equally well as indicated by no differences in protein expressions of myosin heavy chain I and IIa in the myotubes^18^, and by visual examination in the microscope. During the culturing process muscle cells were incubated in a humidified CO_2_ (5%) atmosphere at 37 °C, and medium was changed every two to three days. Experiments were performed on cells from passage number two to four. For each experiment the passage number remained constant.

### Scintillation proximity assay (SPA) and lipolysis assay

Cells were cultured in 96-well Scintiplate^®^ TC (7,000 cells/well), and were allowed to proliferate for seven days and differentiate for six days. Thereafter cells were given fresh DMEM medium without phenol red supplemented with [1-^14^C]oleic acid (0.5 µCi/ml, 100 µM). FA accumulation was monitored regularly over the next 24 h (at 0, 2, 4, 6, 8 and 24 h) using 2450 Microbeta^2^ scintillation counter (PerkinElmer). Thereafter, the cells were washed twice with PBS containing BSA (0.5%) before the cells were incubated in DPBS supplemented with HEPES (10 mM), BSA (0.5%) and glucose (100 µM). The decline in accumulated [1-^14^C]oleic acid was measured over 6 h (at 0, 2, 4 and 6 h) using 2450 Microbeta^2^ scintillation counter in the presence or absence of triacsin C (10 µM), a potent inhibitor of long-chain FA CoA synthetase^58^. Thus, triacsin C inhibited FA recycling into the TAG pool^39^ and FA oxidation^59^, and is as such a measure of total lipolytic activity. Re-esterification of oleic acid was calculated from lipolysis measurements as [triacsin C present (total lipolysis) – triacsin C absent (basal lipolysis)] as reported previously^59,60^. Lipolysis and re-esterification were related to the labelled TAG pool as measured from the lipid distribution assay. After finishing the experiments cells were harvested in NaOH (0.1 M) and protein levels in the cell lysates were measured by the Bio-Rad protein assay using a VICTOR™ *X*4 Multilabel Plate Reader (PerkinElmer). Data were related to total cell protein concentration.

### Lipid distribution

The last 24 h of the differentiation process the myotubes were incubated with [1-^14^C]oleic acid (0.5 µCi/ml, 100 µM). Myotubes were then washed twice with PBS and harvested with two additions of 125 µl distilled water. Cellular lipids were isolated as previously described^61^ by extraction of the homogenized cell fraction, separation of lipids by thin layer chromatography (TLC) and quantification by liquid scintillation (Tri-Carb 1900, PerkinElmer). A non-polar solvent mixture of hexane:diethyl ether:acetic acid (65:35:1) was used to separate the lipids. The amount of lipids was related to total cell protein concentration determined by the Bio-Rad protein assay.

### Isolation of LDs from skeletal muscle cells

Skeletal muscle cells were cultured and differentiated on large petri dishes (10 cm), five dishes per donor. The last 24 h of the differentiation period the muscle cells were stimulated with oleic acid (100 µM). Each dish was washed twice with cold PBS, before all free liquid was removed and cells were harvested in suspension buffer (25 mM tricine pH 7.8, 8.6% sucrose and complete protease inhibitor), mixed gently and frozen at −80 °C.

To disrupt cells, samples were thawed slowly in ice-water slurry for ~30 min and transferred to a precooled N_2_ cell disruption vessel (#4639, Parr Instrument Company). Samples were exposed to N_2_ (600 psi, 20 min) before slowly released through the drain into a 15 ml tube. The disrupted cell lysate were subsequently centrifuged (3,000 × *g*, 10 min at 4 °C) to remove nuclei and undisrupted cell debris. The supernatant containing LDs was adjusted to 2 ml and transferred to the bottom of a Beckman centrifugation tube (#Z30319SCA, Beckman) followed by a second layer consisting of 1.8 ml wash buffer (20 mM HEPES pH 7.4, 100 mM KCl, 2 mM MgCl_2_, 4% sucrose (w/v)), and a top layer of 0.4 ml collection buffer (20 mM HEPES pH 7.4, 100 mM KCl, 2 mM MgCl_2_). Tubes were balanced and centrifuged for 60 min in a SW60Ti rotor at 200,000 × *g* at 4 °C in a XL-90 Ultracentrifuge (Beckman). The top layer (~0.4 ml) was isolated with a tube slicer prior to collecting of floating LDs. 30 µl was taken to morphology check on a fluorescence microscope (Olympus IX81, Tokyo, Japan) after staining with Bodipy 493/503 (5 µM). Pictures were taken at 40x magnification, two pictures of each sample, and images were analysed using ImageJ version 1.50i (NIH, US). Measurement of TAG mass in isolated LDs was performed with the TG PAP 150-kit according to the supplier’s protocol.

### Imaging of myotubes

Skeletal muscle cells were cultured on glass bottom micro well dishes. The last 24 h of the differentiation period some parallels were treated with 100 µM oleic acid. Cells were fixed with PFA (4%) in NaPi (0.1 M) for 1 h at room temperature, and were stored in NaPi (0.1 M at 4 °C) before staining. Staining of fixed cells was done with Bodipy 493/503 (1 µM) to stain for lipid droplets plus Hoechst 33342 (5 µM) to stain for nuclei, both for 30 min. Pictures were taken at 40x magnification, five pictures of each cell sample, using a confocal microscope (LSM 710, Zeiss, Oberkochen, Germany). Images were analysed using Image J version 1.50i (NIH, US).

### Lipid composition of isolated LDs

After isolation of LDs, samples were analysed for TAGs, glycerophosphocholines, glycerophosphoethanolamines, and CEs using high performance liquid chromatography coupled to time-of-flight mass spectrometry. In addition, analysis of total FA profiles was performed by acid hydrolysis, transmethylation and gas chromatography with flame ionization detector. 80 µl of each sample was added 800 µl Folch extraction solution and mixed thoroughly before 200 µl dH_2_O was added. The organic layer was removed and evaporated to dryness and reconstituted in 80 µl dichloromethane:isopropanol (1:3) prior to injection. The lipid analyses were performed by Vitas AS (Oslo, Norway).

### Complete FA oxidation – CO_2_ formation

Skeletal muscle cells (7,000 cells/well) were cultured on 96-well CellBIND^®^ microplates. [1-^14^C]oleic acid (0.5 µCi/ml, 100 or 400 µM) were given during 4 h trapping as described^62^ with or without 24 h prelabelling. In brief, a 96-well UniFilter^®^-96 GF/B micro plate was mounted on top of the CellBIND^®^ plate and CO_2_ production was measured in DPBS medium with HEPES (10 mM) and L-carnitine (1 mM) adjusted to pH 7.2-7.3. CO_2_ production was assessed using a 2450 MicroBeta^2^ scintillation counter (PerkinElmer). Data were related to total cell protein concentration measured in the cell lysates by the Bio-Rad protein assay.

### Incomplete FA β-oxidation – ASMs

After 4 or 24 h incubation with [1-^14^C]oleic acid (0.5 µCi/ml, 100 or 400 µM) at day 6-7 of differentiation, measurement of ASMs was performed using a method modified from Skrede *et al*.^63^ and described by Bakke *et al*.^59^. In short, 30 µl incubation media were transferred to a Nunc 96-well polystyrene conical bottom micro well plate, precipitated with 10 µl BSA (6%) and 100 µl ice-cold HClO_4_ (1 M), and centrifuged at 2,100 × *g* for 10 min at 4 °C. Thereafter, 30 µl of the supernatant was counted by liquid scintillation. ASMs mainly consist of tricarboxylic acid cycle metabolites and reflect incomplete FA β-oxidation in the mitochondria. Data were related to total cell protein concentration measured in the cell lysates by the Bio-Rad protein assay.

### RNA isolation and analysis of gene expression by real-time qPCR

Skeletal muscle cells were cultured and differentiated in 25 cm^2^ NUNC flasks. Total RNA was isolated using RNeasy Mini Kit according to the supplier’s protocol. RNA was reversely transcribed with a High-Capacity cDNA Reverse Transcription Kit and TaqMan Reverse Transcription Reagents using a PerkinElmer Thermal Cycler 9600. Primers were designed using Primer Express^®^ and qPCR was performed using an ABI PRISMT 7000 Detection system (Thermo Fisher Scientific)^52^. Expression levels were normalized to the housekeeping gene acidic ribosomal phosphoprotein P0 (*RPLP0*). Glyceraldehyde-3-phosphate dehydrogenase (*GAPDH*) was also analysed as housekeeping gene; there were no differences between normalizing for *RPLP0* or *GAPDH*. The following forward and reverse primers were used at concentration of 30 µM: *ATP5F1A* (acc. no.: NM_001001937.1), *ATP5MC2* (acc. no.: NM_001002031.2), *CD36* (acc. no.: L06850), *COX4I1* (acc. no.: NM_001861.4), *COX5B* (acc. no.: M19961.1), *CPT1A* (acc. no.: L39211), *CPT1B* (acc. no.: D1852C12), *DGAT1* (acc. no.: NM012179), *DGAT2* (acc. no.: NM032564), *FABP4* (acc.no.: J02874), *GAPDH* (acc. no.: NM002046), *G0S2* (acc. no.: M69199.1), *NDUFA8* (acc. no.: NM_014222.2), *NDUFS1* (acc. no.: NM_005006.6), *PLIN2* (acc. no.: NM001122), *PLIN3* (acc. no.: AF057140), *PPARD* (acc. no.: BC002715), *PPARGC1A* (acc. no.: NM013261.3), *RPLP0* (acc. no.: M17885), *SDHB* (acc. no.: NM_003000.2), *UQCR11* (acc. no.: NM_006830.3), and *UQCRB* (acc. no.: NM_006294.4).

### Immunoblotting

Myotubes were harvested in Laemmli buffer (0.5 M Tris-HCl, 10% SDS, 20% glycerol, 10% β-mercaptoethanol, and 5% bromophenol blue). The proteins were electrophoretically separated on Mini-Protean^®^ TGX™ gels (4-20%) with Tris/glycine buffer (pH 8.3) followed by blotting to nitrocellulose membrane and incubation with antibody against HSL (polyclonal, raised in rabbit, 1:200), HSL phosphorylated at serine 660 (HSL^Ser660^, polyclonal, raised in rabbit, 1:1,000), ATGL (polyclonal, raised in rabbit, 1:200), PLIN2 (polyclonal, raised in rabbit, 1:1,000), PLIN3 (polyclonal, raised in rabbit, 1:1,000), and β-actin (monoclonal, raised in rabbit, 1:5,000). Immunoreactive bands were visualized with enhanced chemiluminescence (Chemidoc XRS, Bio-Rad) and quantified with Image Lab (version 4.0) software. All samples were derived at the same time and processed in parallel. Expression levels were normalized to one sample used as loading control and to the housekeeping protein β-actin. Complete immunoblots are reported in Supplementary Fig. S2. We were not able to find antibodies with sufficient level of quality to measure protein expressions of DGATs, CPT1s or PGC1α.

### Presentation of data and statistics

Data are presented as means ± SEM in nmol/mg cell protein unless stated otherwise. The value *n* represents the number of different donors, each with at least duplicate observations. Statistical analyses were performed using GraphPad Prism 6 for Windows (GraphPad Software, Inc., La Jolla, CA, US) or SPSS version 22 (IBM^®^ SPSS^®^ Statistics for Macintosh, Armonk, NY, US). Two-tailed Wilcoxon matched-pairs signed rank test was used within groups, whereas two-tailed Mann-Whitney test was used to evaluate effects between groups. Linear mixed-model analysis was used to compare differences between conditions with within-donor variation and simultaneously compare differences between groups with between-donor variation. The linear mixed-model analysis includes all observations in the statistical analyses and takes into account that not all observations are independent. A *p*-value < 0.05 was considered significant.

## Electronic supplementary material

Supplementary Figures

## Data Availability

The datasets generated during and/or analysed during the current study are available from the corresponding author on reasonable request.
