# Supplementary material for: Higher lipid turnover and oxidation in cultured human myotubes from athletic versus sedentary young male subjects
Source: Sci Rep. 2018 Dec 3;8:17549. doi: 10.1038/s41598-018-35715-7 (PMC6277406; doi:10.1038/s41598-018-35715-7)
Supplement: Supplementary file 1 — Supplementary Figures [file 41598_2018_35715_MOESM1_ESM.pdf]

Higher lipid turnover and oxidation in cultured human myotubes from athletic versus sedentary young male subjects

Jenny Lund[1*], Siw A. Helle[1], Yuchuan Li[2], Nils G. Løvsletten[1], Hans K. Stadheim[3], Jørgen Jensen[3], Eili T. Kase[1], G. Hege Thoresen[1,4], Arild C. Rustan[1]

[1]Department of Pharmaceutical Biosciences, School of Pharmacy, University of Oslo, Oslo, Norway

[2]Department of Nutrition, Institute of Basic Medical Sciences, University of Oslo, Oslo, Norway

[3]Department of Physical Performance, Norwegian School of Sport Sciences, Oslo, Norway

[4]Department of Pharmacology, Institute of Clinical Medicine, University of Oslo, Oslo, Norway

**Corresponding author*

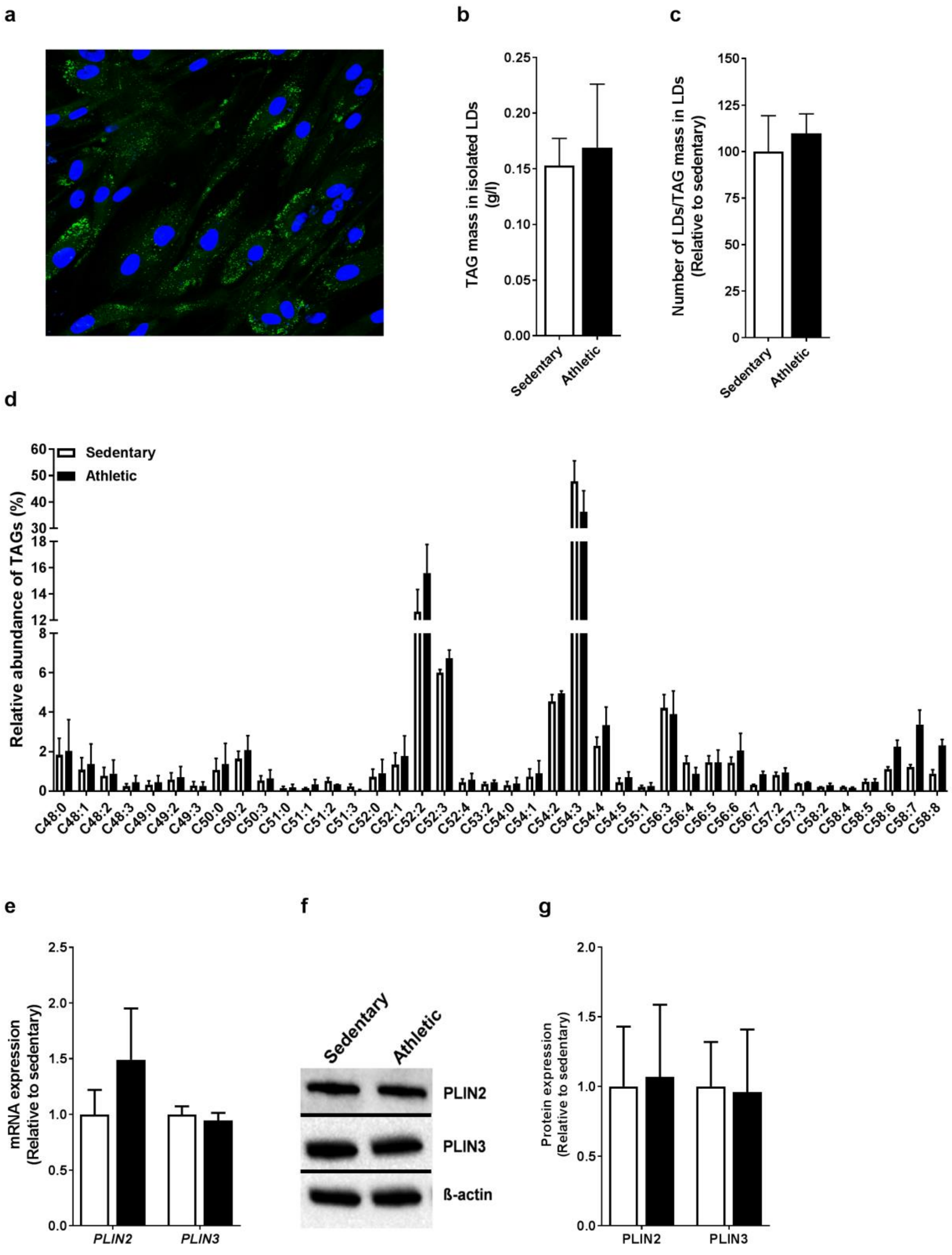

Supplementary Figure S1. Lipid droplets characteristics and expression of PLINs. Satellite cells isolated from biopsies from *musculus vastus lateralis* from athletic and sedentary subjects were cultured and differentiated into myotubes. The last 24 h of the differentiation period the myotubes were treated with oleic acid (100 µM). Samples were harvested for lipid droplet (LD) isolation. **(a)** One representative image of myotubes after fixation and staining for 30 min with Bodipy 493/503 (green) and Hoechst 33342 (blue) for LDs and nuclei, respectively. **(b)** Triacylglycerol (TAG) mass in isolated LDs. Data are presented as means ± SEM (n = 3 in each group) in g/l. **(c)** Number of isolated LDs was quantified using Image J, and TAG mass was measured in the isolated LD lysates. Number of isolated LDs was then related to the TAG mass in the isolated LDs. Data are presented as means ± SEM (n = 3 in each group) relative to sedentary. **(d)** Lipid composition analysis of isolated lipid droplets from myotubes using high performance liquid chromatography coupled to time-of-flight mass spectrometry. Data are presented as means ± SEM (n = 3 in each group) in relative abundance. **(e)** RNA was isolated and mRNA reversely transcribed before expressions of perilipin (*PLIN*) *2* and *PLIN3* were assessed by qPCR. Values are presented as means ± SEM (n = 6 in each group), and corrected for the average of the housekeeping gene acidic ribosomal phosphoprotein P0 (*RPLP0*). The results were normalized to the results of the mRNA expression for myotubes from sedentary subjects. **(f and g)** Protein expressions of PLIN2 and PLIN3 were analysed by immunoblotting of protein isolated from cell lysates. f, representative immunoblots. g, quantified expressions of the proteins. All values were corrected for the housekeeping control β-actin, and are presented as means ± SEM (n = 6 in each group). All samples were derived at the same time, from the same experiment, and processed in parallel. Full-length blots are presented in Supplementary Figure S2.

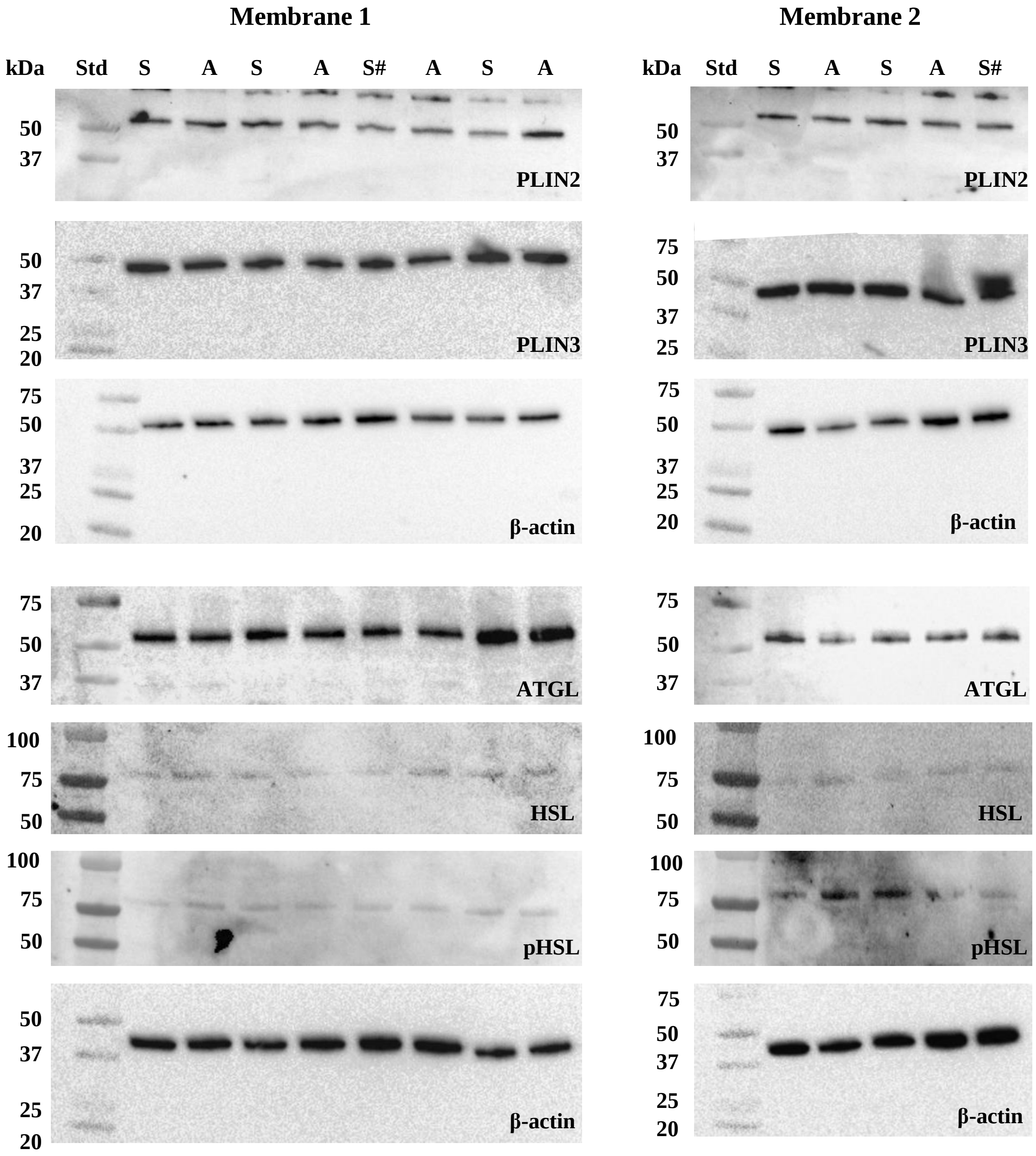

Supplementary Figure S2. Unadjusted immunoblots of PLIN2, PLIN3, ATGL, HSL, phospho-HSL^{Ser660}, and β-actin. Complete immunoblots, showing samples from all sedentary (S) and athletic (A) donors, merged with standards (Std). One sample was used as loading control (#). The PLIN2 antibody is a polyclonal antibody and the band is located at ~55 kD. There is an unspecific band at ~70 kD that probably is heavy chain. The PLIN3 antibody also is a polyclonal antibody and the band is located at ~52 kD. Membranes for PLIN2 and PLIN3 were cut approximately between 75 and 100 kDa to be able to use the top half for incubation with another antibody with higher molecular weight (not included here). The ATGL antibody is a polyclonal antibody and the band is located at 54 kD. The β-actin antibody is a monoclonal antibody used as housekeeping protein and the band is located at 45 kDa. First β-actin blot was used as housekeeping for PLIN2 and PLIN3, whereas the second β-actin blot was used as housekeeping for ATGL and HSL. Both the HSL antibody and the phospho-HSL^{Ser660} antibody are polyclonal antibodies that consist of two bands at 81 and 83 kD. There is an unspecific weaker band at ~52 kD that also is reported by producer but it is unknown what it is.
